# *Racgap1* knockdown results in cells with multiple cilia due to cytokinesis failure

**DOI:** 10.1111/ahg.12529

**Published:** 2023-09-28

**Authors:** Basudha Basu, Alice V. R. Lake, Becky China, Katarzyna Szymanska, Gabrielle Wheway, Sandra Bell, Ewan Morrison, Jacquelyn Bond, Colin A. Johnson

**Affiliations:** ^1^ Division of Molecular Medicine, Leeds Institute of Medical Research University of Leeds Leeds UK; ^2^ University Hospital Southampton NHS Foundation Trust Southampton UK; ^3^ Faculty of Medicine, Human Development and Health University of Southampton Southampton UK

**Keywords:** centrioles, cilia, ciliopathies, cytokinesis

## Abstract

Most mammalian cells have a single primary cilium that acts as a signalling hub in mediating cellular functions. However, little is known about the mechanisms that result in aberrant supernumerary primary cilia per cell. In this study, we re‐analysed a previously published whole‐genome siRNA‐based reverse genetic screen for genes mediating ciliogenesis to identify knockdowns that permit multi‐ciliation. We identified siRNA knockdowns that caused significant formation of supernumerary cilia, validated candidate hits in different cell‐lines and confirmed that RACGAP1, a component of the centralspindlin complex, was the strongest candidate hit at the whole‐genome level. Following loss of RACGAP1, mother centrioles were specified correctly prior to ciliogenesis and the cilia appeared normal. Live cell imaging revealed that increased cilia incidence was caused by cytokinesis failure which led to the formation of multinucleate cells with supernumerary cilia. This suggests that the signalling mechanisms for ciliogenesis are unable to identify supernumerary centrosomes and therefore allow ciliation of duplicated centrosomes as if they were in a new diploid daughter cell. These results, demonstrating that aberrant ciliogenesis is de‐coupled from cell cycle regulation, have functional implications in diseases marked by centrosomal amplification.

## 1 INTRODUCTION

Most mammalian cells, with the exception of some specialised cells, have a single primary cilium that is essential for normal cellular function (Malicki & Johnson, 2017; Satir et al., 2010; Wheway et al., 2018). Primary cilia are non‐motile organelles with a so‐called ‘9+0’ arrangement of microtubules that comprise the axoneme. They therefore lack the central pair of microtubules present in ‘9+2’ motile cilia and act as sensors and transducers of signals and play important roles in growth and development. Disruption or loss of the primary cilium can cause a range of disorders called ciliopathies. While most disease‐causing variations cause a loss of cilia or ciliary function, the gain of supernumerary primary cilia also results in defects due to a reduction in ciliary signalling capacity in each individual cilium (Mahjoub & Stearns, 2012). In cells with multiple primary cilia, pathways such as Sonic Hedgehog (SHH) are affected due to ciliary dilution of available Smo molecules. This phenomenon has also been observed for other signalling proteins that localise to cilia such as 5‐hydroxytryptamine (serotonin) receptor 6 (5‐HTR6) or ARL13B (Mahjoub & Stearns, 2012). Indeed, there are some documented ciliopathy phenotypes with supernumerary cilia in affected tissues. Foetal kidney cysts from Meckel–Gruber syndrome patients with *MKS1* or *TMEM67* variations have supernumerary centrosomes, spindle poles and primary cilia in addition to significantly increased ciliary length (Tammachote et al., 2009). The loss of PKD1 in mice is a model of autosomal dominant polycystic kidney disease, and while it is marked by dramatic centrosome amplification, it is not known if this leads to supernumerary cilia or if this could contribute to disease mechanism (Battini et al., 2008). However, targeted resorption of cilia has been suggested to be a possible therapeutic intervention for autosomal dominant polycystic kidney disease that could reduce cyst formation and slow disease progression (Nikonova et al., 2018). As an exception to the rule, normal choroid plexus epithelial cells, which produce cerebrospinal fluid in the brain ventricles, display clusters of up to two dozen non‐motile (9+0) primary cilia (Narita et al., 2010). These are distinct and separate from the mature ependyma that forms hundreds of (9+2) motile cilia (Narita & Takeda, 2015), although interestingly, both these ciliary types share the *FOXJ1* pathway used in motile cilia biogenesis (Narita et al., 2012).

Aberrant centriole numbers and centrosome amplification are also observed in many cancers (Chan, 2011; Godinho & Pellman, 2014; Pihan et al., 1998) but paradoxically, renal, pancreatic and breast cancers have reduced cilia incidence or loss of cilia (Basten et al., 2013; Seeley et al., 2009). However, there are exceptions, and it has been reported that SHH‐related cancers can either be driven or inhibited by the presence of cilia depending on whether the variation is in *SMO* or *GLI2*, respectively (Han et al., 2009; Wong et al., 2009). In addition, the presence of cilia in the cancer tissue of patients with pancreatic ductal adenocarcinoma (PDA) correlated with an elevated frequency of lymph node metastasis compared to those PDA patients with cancers lacking cilia (Emoto et al., 2014).DNA damage caused by ionising radiation can also give rise to cancer‐like phenotypes and the early stages of these are marked by cilia amplification (Conroy et al., 2012; Filipova et al., 2015).

In order to identify potential regulators of cilia number, we designed an unbiased reverse genetics strategy. We queried the raw dataset of a previously published reverse genetics screen that identified genes required for ciliogenesis or cilia maintenance (Wheway et al., 2015) and re‐analysed to identify knockdowns that caused more than one primary cilium to develop per cell (that we term ‘supernumerary primary cilia’). We confirmed three top hits in a secondary screen, for genes that interact and are involved in late mitosis stages. The top candidate was *Racgap1*, an essential regulator of cytokinesis and component of the centralspindlin complex (Baumann, 2013; Kim et al., 2014; Lekomtsev et al., 2012). *RACGAP1* has been found to be overexpressed in multiple cancers (Yang et al., 2018) and is often associated with strong cancer progression and invasion (Imaoka et al., 2015; Saigusa et al., 2015). However, *RACGAP1* has not previously been associated with supernumerary cilia, suggesting that it has a unique regulatory function in coupling mitotic progression with correct ciliogenesis.

## 2 MATERIALS AND METHODS

### 2.1 Reanalysis of whole‐genome reverse genetics screen data to identify genes increasing the incidence of supernumerary cilia

**FIGURE 1:**
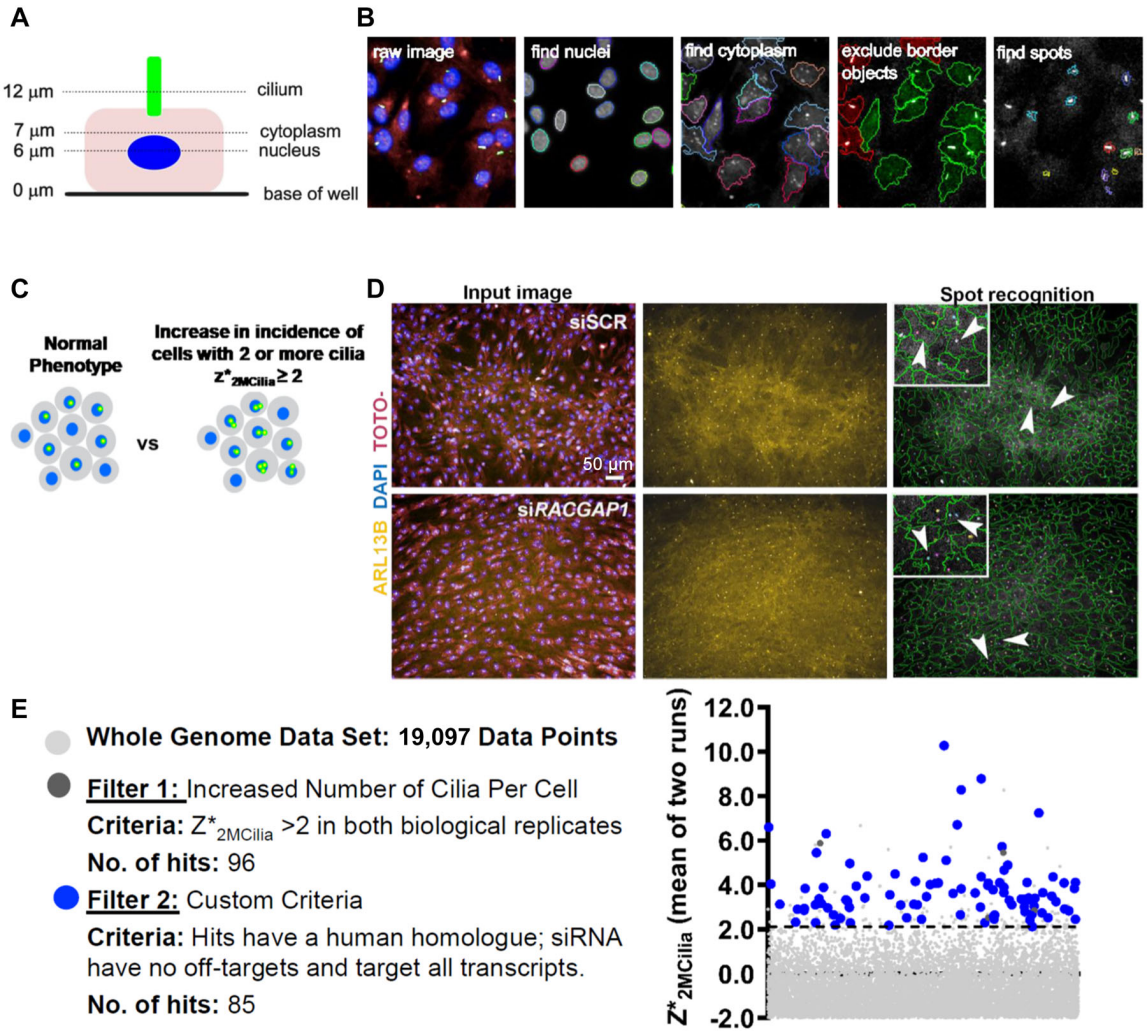
Re‐analysis of a whole‐genome cilia screen to identify genes that suppress supernumerary cilia. (a) Schematic of a cell showing the focal planes used to image nuclei (blue), cytoplasm (pink) and ciliary axonemes (green). (b) hTERT‐RPE1 cells, immunolabelled with anti‐Arl13b to mark cilia (green) and stained with DAPI and TOTO‐3 to detect the nucleus and cytoplasm, respectively, imaged using an Operetta high‐content imaging system, showing representative images from Harmony/Columbus software of cilia recognition (‘find spots’). (c) Schematic of the original analysis and re‐analysis of the cilia screen dataset. The parameters were readjusted to identify cells with two or more cilia (green). (d) Spot recognition protocol showing identification of cells with two cilia (stained for Arl13b; gold) for mIMCD3 cells counterstained for nuclei (DAPI; blue) and cytoplasm (TOTO‐3; magenta). Supernumerary cilia are indicated (white arrowheads) with detail shown in magnified insets. (c) Filtration criteria for identifying the 85 genes that were taken forward into a secondary screen from the whole‐genome reverse genetics screen dataset for average robust *z‐*scores of increases in two or more cilia (z*_2MCilia_). Candidate hits are identified by blue points in the scatter plot.

We used an image recognition protocol that allows high‐content analysis of images and permits rapid identification and quantification of cells with 0, 1, or >1 cilia (Figure 1a,b). We used the data columns that showed the fraction of cells per well that had two or more cilia and analysed that to generate robust *z*‐scores for the two biological replicates (‘runs’) of the primary screen. The robust *z*‐score is a function of the median of a population and the median absolute deviation (MAD) (Zhang, 2011). This is calculated as z* = |*x* – MED(*x*)|/MAD(*x*), where z* = robust *z*‐score, MED(*x*) is the median and MAD is the median absolute deviation. The final set of 85 genes were analysed to check which Gene Ontology cellular components, KEGG pathways and Reactome pathways (Fabregat et al., 2017) were enriched.

### 2.2 Cell culture and transfection

Human hTERT‐RPE1 and mouse mIMCD3 cell lines were grown in DMEM/F12 – GlutaMAX™ supplemented with 10% foetal bovine serum (FBS). mIMCD3 cells stably transfected with 5‐HT6‐GFP, and LifeAct‐GFPmIMCD3 cells were gifts of Prof. Ronald Roepman (Radboud University Medical Centre, Nijmegen) and Dr. Georgia Mavria (University of Leeds). Cells were cultured under conditions of 37°C and 5% CO_2_. hTERT‐RPE1 cells were plated on matrigel‐coated coverslips and reverse‐transfected with siRNAs (SMARTpool ON‐TARGETplus siRNAs or siGENOME siRNAs; Dharmacon Inc.) against mouse or human *Racgap1* or scrambled control (83 nM final concentration). The knockdown was performed in OptiMEM with 0.2% FBS in order to promote ciliogenesis and cells were fixed after 72 h. All the siRNA sequences are listed in Tables S2 and S3.

### 2.3 Serum treatment of cells

Cells were treated with siRNA as normal in OptiMEM +0.2% serum to knockdown *Racgap1*. A subset of cells were serum starved for the entire 72 h, while other cells had 10% FBS added for the final 24 h to induce cell cycling. The cells were then fixed in ice‐cold methanol for immunolabelling and imaging.

### 2.4 Immunolabelling

Cells were washed in phosphate‐buffered saline (PBS) with 0.1 mM Ca^2+^ and Mg^2+^ and fixed in ice‐cold methanol at −20°C for 5 min. The cells were blocked and stained with a subset of the following antibodies at 4°C overnight: anti‐ARL13B (Proteintech, 1:1000), anti‐γ‐tubulin (Proteintech, 1:1000), anti‐polyglutamylated tubulin (clone GT‐335, Sigma, 1:1000) and CEP164 (1:50,000). Following washes, species‐specific secondary antibodies (Life Technologies, 1:2000) were added for 90 min followed by washing in PBS+0.05 Triton‐X‐100 and PBS. The coverslips were then stained with DAPI and mounted. The anti‐CEP164 monoclonal antibody was a generous gift from Dr. Ciaran Morrison (National University of Ireland Galway).

### 2.5 High‐content imaging and analysis

We used a previously published protocol (Wheway et al., 2015) to image and analyse cells for multiple cilia. Briefly, we used mIMCD3 or hTERT‐RPE1 cells to assay for a supernumerary cilia phenotype 72 h after transfection, by imaging at three focal planes to detect for cilia (Arl13b), nucleus (DAPI) and cytoplasm (TOTO‐3) (Figure 1a). We then used optimised spot detection algorithms in ‘Columbus’ software (PerkinElmer) to recognise cilia.

### 2.6 Live imaging

Cells were imaged live on a Nikon Biostation IM at 37°C in 10 mM HEPES‐buffered medium (pH 7.2) with no phenol red. Images were collected every 5 min from six image areas each per treatment for a duration of 24 h. Images were processed and analysed using FIJI.

### 2.7 Fluorescence microscopy

A Zeiss ‘ApoTome’ microscope was used to image fluorescently stained coverslips at 63× magnification. Images were processed and analysed using FIJI.

### 2.8 Semi‐quantitative reverse transcription‐polymerase chain reaction

Cells were treated with siRNA for 72 h before whole RNA was extracted and cDNA was generated following the manufacturer's instructions (SuperScript III Reverse Transcriptase; Thermofisher Scientific). We used human siRNA for hTERT‐RPE1 and with mouse‐specific siRNA for mIMCD3 cells (Tables S2 and S3). Reverse transcription‐polymerase chain reaction (RT‐PCR) was run for 50 cycles and the band intensities were measured using ImageLab. The band intensities for *RACGAP1* were normalised to *GAPDH* band intensity values from the same sample.

### 2.9 Quantification and statistical analysis

Quantification of cilia numbers and centrosomes was done manually using FIJI. All Operetta images were analysed with the Columbus software (Perkin Elmer), and one‐way ANOVA with Fisher's least significant difference was carried out for all the datasets. Robust *z*‐scores were used for the primary and secondary screens and a cut‐off of +2 was used to identify genes that cause significant increase in multiple cilia (+2 correspond to an increase with a *p* < 0.05).

### 2.10 Data availability

Screen datasets are available at the following site: https://doi.org/10.5518/1044.

## 3 RESULTS

### 3.1 Knockdown of 85 genes increases the number of cells with supernumerary cilia

A previous genome‐wide screen identified genes involved in ciliogenesis and cilia maintenance (Wheway et al., 2015) using the Operetta high‐content imaging system and Columbus software workflow (Figure 1a,b). The screen identified genes which, when knocked‐down, resulted in loss of cilia in the ciliated mouse inner medullary collecting duct (mIMCD3) cell line. We took the raw data from that screen and re‐analysed it to identify genes which, when knocked‐down, resulted in supernumerary cilia (>1 cilium) in both ‘runs’ of the primary screen (Figure 1c,d). We selected knockdowns that resulted in robust *z‐*scores (referred to as z*_2MCilia_) of >+2 (which represent a *p* value of <0.05 for increase in two or more cilia). These 96 genes were then filtered using the following criteria: (i) knockdowns that did not cause a significant phenotype in both biological replicates of the primary screen were excluded; (ii) genes were excluded if they did not have ‘on‐target siRNAs’ that targeted all annotated transcripts of that gene; and (iii) mouse genes for which there was no clear human orthologue were also excluded. From an initial 19,097 data points identified from the primary screen, 85 genes met these criteria (Figure 1e and Table S1).

Network analysis using the Search Tool for Recurring Instances of Neighbouring Genes (STRING https://string‐db.org/) (Jensen et al., 2009; Szklarczyk et al., 2019) identified a central network of cell cycle and G2‐M transition genes such as *Ccnb1* and *Cdk1* (Table S1 and Figure S1). More peripheral nodes were involved in DNA replication (*Prim2, Ssrp1*), structural constituents of ribosomes (*Rps2*, *Rps20*, *Rps7*) and the proteasome (*Psma7, Pomp*). The gene list was enriched in cell cycle genes, comprising 8% (7/85) (*q*‐value = 5.868 × 10^−8^, false discovery rate = 0.0003 with Benjamini–Hochberg correction when compared to the whole genome)

### 3.2 Secondary screen to validate genes that increase the incidence of supernumerary cilia

**FIGURE 2:**
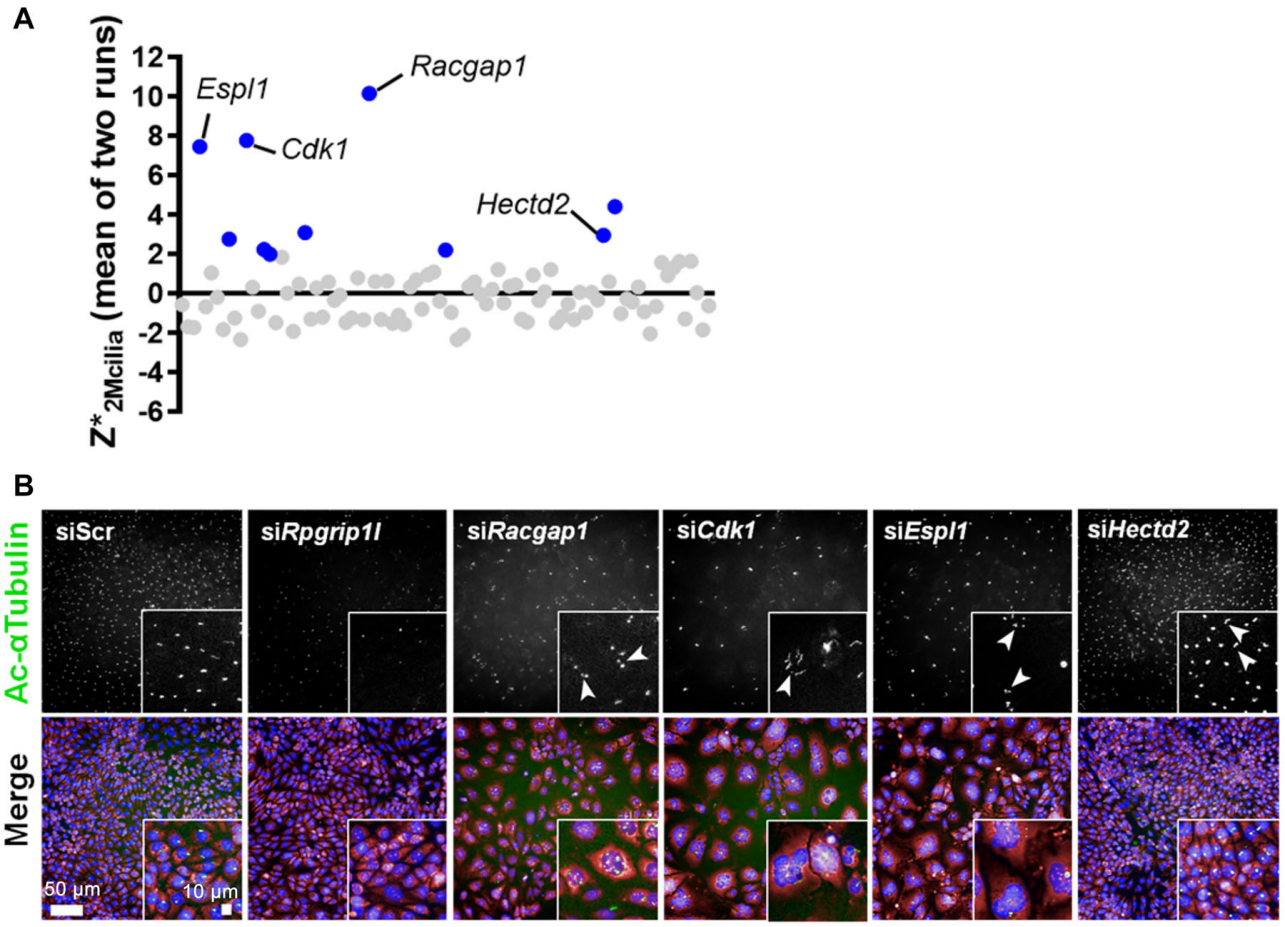
Validated hits from the secondary screen for increased supernumerary cilia. (a) The average robust *z*‐scores from two biological replicates (runs) for incidence of cells with two or more cilia (z*_2MCilia_) are plotted, with the top 10% of hits identified by blue points in the scatter plot. The top four hits and their z*_2MCilia_ values are *Racgap1* (10.141), *Cdk1* (7.769), *Espl1* (7.455) and *Hectd2* (2.940). (b) Raw image data of top hits from the secondary screen to identify supernumerary cilia. Primary cilia are visualised by staining for acetylated α‐tubulin (green), with cells counterstained for nuclei (DAPI; blue) and cytoplasm (TOTO‐3; magenta). Scale bar = 50 μm. Supernumerary cilia are indicated (white arrowheads) with detail shown in magnified insets.

The 85 genes identified were taken forward in a secondary validation screen performed using a ‘Smartpool’ of four different modified siRNAs (Dharmacon ON‐Target Plus siRNA). These were of a different chemistry to the siRNA that was used in the primary screen (Dharmacon siGENOME) in mIMCD3 cells. These were chosen as a control to make sure that similar effects on cilia number were seen even with siRNAs of different chemistries. si*RPGRIP1L* was used as a transfection control as it causes cilia loss, while scrambled negative control siRNA (siScr) was used as a negative control. After qualitative and statistical analysis to calculate z*_2MCilia_ scores, 10 hits (Table 1) were validated to reproducibly and significantly increase the incidence of supernumerary cilia (average z*_2MCilia_ >+2; Figure 2a), comprising an overall validation rate of 11.2%. The validation dataset had three out of 77 negative controls with average z*_2MCilia_ >+2 and the screen therefore had a false positive rate of 3.9%. There were three top hits (*Cdk1*, *Espl1* and *Racgap1*) that could be taken forward for further investigation, as they all had z*_2MCilia_ >7.45 (equivalent to *p* < 0.00001). These hits also had a clear qualitative difference from the negative controls when assessing raw image data (Figure 2b). Cell numbers were significantly reduced (Table 1) and raw image data showed a high proportion of large, multinucleated cells and decreased cell number, suggesting cell division defects following knockdown of these genes. *Cdk1*, *Espl1* and *Racgap1* encode proteins that interact. Cdk1 phosphorylates both Espl1 and Racgap1, thus highlighting a functional network or pathway that, when perturbed, caused the increased incidence of supernumerary cilia. Only one of the 10 validated hits did not cause a statistically significant decrease in cell number: si*Hectd2* treatment resulted in average z*_2Mcilia_ of 2.94 and average z*_cell_ (robust *z*‐score for cell number) of 0.775.

**TABLE 1:** Validated hits from the increase supernumerary cilia secondary screen.

		Primary screen			Secondary screen		
Gene	Transcript Ref Seq	Average z*_cell_	Average z*_cilia_	Average z*_2Mcilia_	Average z*_cell_	Average z*_cilia_	Average z*_2Mcilia_
Racgap1	NM_012025	–8.045	–7.278	**3.784**	–14.965	–9.764	**10.141**
Cdk1	NM_007659	–8.621	–4.202	**5.881**	–13.243	–8.713	**7.769**
Espl1	NM_001014976	–7.388	–5.550	**3.939**	–14.877	–3.625	**7.455**
BC089491	NM_175033	–7.791	–8.076	**3.842**	–4.101	–0.828	**4.399**
Tfdp1	NM_009361	–5.820	–3.764	**7.238**	–9.486	–0.947	**3.086**
Hectd2	NM_172637	0.030	–0.595	**3.548**	0.775	2.138	**2.940**
Ssrp1	NM_182990	–6.993	–1.750	**3.411**	–4.041	–0.778	**2.754**
Lhb	NM_008497	–2.600	–1.557	**3.129**	–6.332	–0.474	**2.233**
Narf	NM_026272	–4.912	–7.298	**4.078**	–2.962	–0.714	**2.195**
Mcm7	NM_008568	–4.501	–3.652	**5.240**	–4.349	–2.646	**1.988**

*Note*: The table shows the average *z*‐scores from two biological replicates for incidence of cells with two or more cilia (z*2Mcilia; values shown in bold). Hits are ordered from highest to lowest.

### 3.3 RACGAP1 depletion increases the incidence of supernumerary cilia in hTERT‐RPE‐1 cells

**FIGURE 3:**
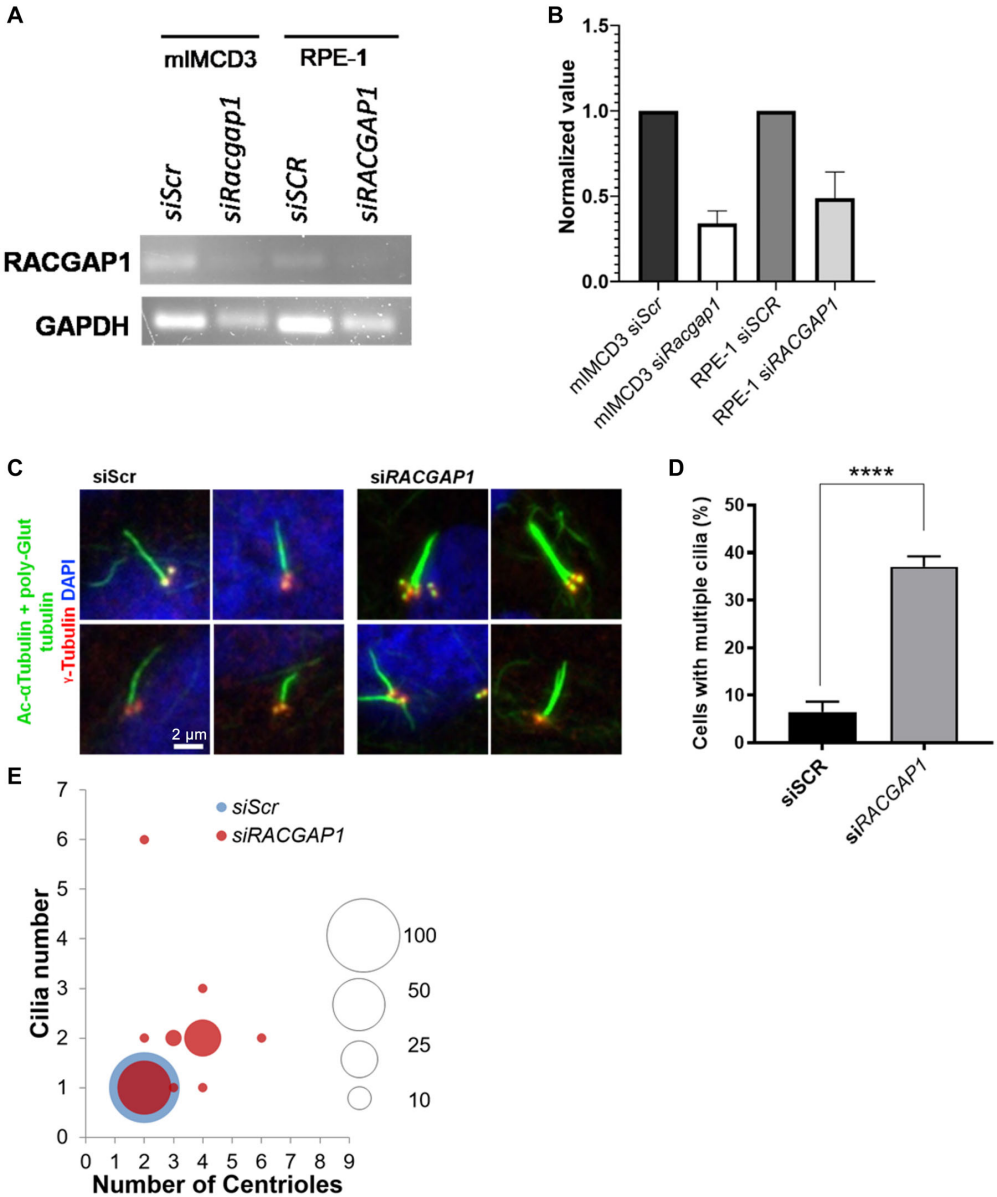
RACGAP1 knockdown results in supernumerary cilia and centrosomes in RPE‐1 cells. (a) Semi‐quantitative RT‐PCR showing reduced expression of *Racgap1* transcripts in both mouse mIMCD3 and human hTERT‐RPE1 cells following knockdown with the cognate, species‐specific siRNAs. (b) The normalised levels of *Racgap1* RT‐PCR bands compared to GAPDH loading control are indicated for siRNA knockdown in mIMCD3 and RPE‐1 cells. (c) Representative primary cilia in hTERT‐RPE1 cells treated with siRNA targeting human *RACGAP1* (si*RACGAP1*) and scrambled negative control siRNA (siScr). Primary cilia are visualised by staining with a combination of acetylated a‐tubulin and poly‐glutamylated tubulin (green), with basal bodies stained for g‐tubulin (red). The negative control siRNA (siScr) cells have a single primary cilium originating from one centriole, whereas si*RACGAP1*‐treated cells have several and accompanied by supernumerary centrioles. Scale bar = 2 mm. (d) Analysis quantifying percentage of cells showing multiple cilia in fields of view (63× magnification) of siSCR‐ and siRACGAP1‐treated hTERT‐RPE1 cells. Total fields of view >30 (>200 cells). *N* = 4 replicates. (e) Bubble graph to quantitate the percentage of cells with different cilia and centriole numbers. The size of the bubble represents the percentage, as indicated in the legend. The normal ratio of cilium to centrioles is 1:2 as indicated for siScr‐treated cells. All other ratios in si*RACGAP1*‐treated cells (red) are aberrant.

We decided to further investigate the topmost ranking hit, *RACGAP1*, for involvement in cilia number control. RACGAP1 along with KIF23 constitutes a heterotetramer motor complex, called the centralspindlin complex that functions in cytokinesis (Baumann, 2013; Kim et al., 2014) and results in the formation of two daughter cells. We examined the effects on cellular and ciliary phenotypes using structured illumination microscopy following siRNA‐mediated knockdown of *RACGAP1* in human hTERT‐RPE1 cells. We confirmed that the gene was successfully knocked‐down by siRNA using semi‐quantitative RT‐PCR (Figure 3a,b). When hTERT‐RPE1 cells were treated with *siRACGAP1*, there was a significant increase in the number of cells with supernumerary cilia compared to cells treated with scrambled negative control siRNA (siScr; Figure 3c,d). Cilia originated from centrioles of separate centrosomes and there was no bifurcation or splitting of the axonemes. Cells treated with *siRACGAP1* also had more centrosomes compared to controls. Control cells had a 1:2 ratio of cilia to centrioles, whereas *siRACGAP*1‐treated cells had additional aberrant ratios (Figure 3e). The most frequent aberrant ratio composed of a 2:4 duplication of both cilia and centrioles. Other abnormal ratios were also observed, such as 2:6, 2:2 and 3:4 that may indicate errors of centriole maturation or imaging artefacts. Nearly 40% of cells had either an increase in centriole number, an increase in cilia incidence, or both, following *RACGAP1* knockdown. In the raw secondary screen data (Figure 2b), si*RACGAP1*‐treated cells showed an increase in cell size compared to control cells (Figure S2B).

### 3.4 Mother centrioles are correctly specified in supernumerary cilia

**FIGURE 4:**
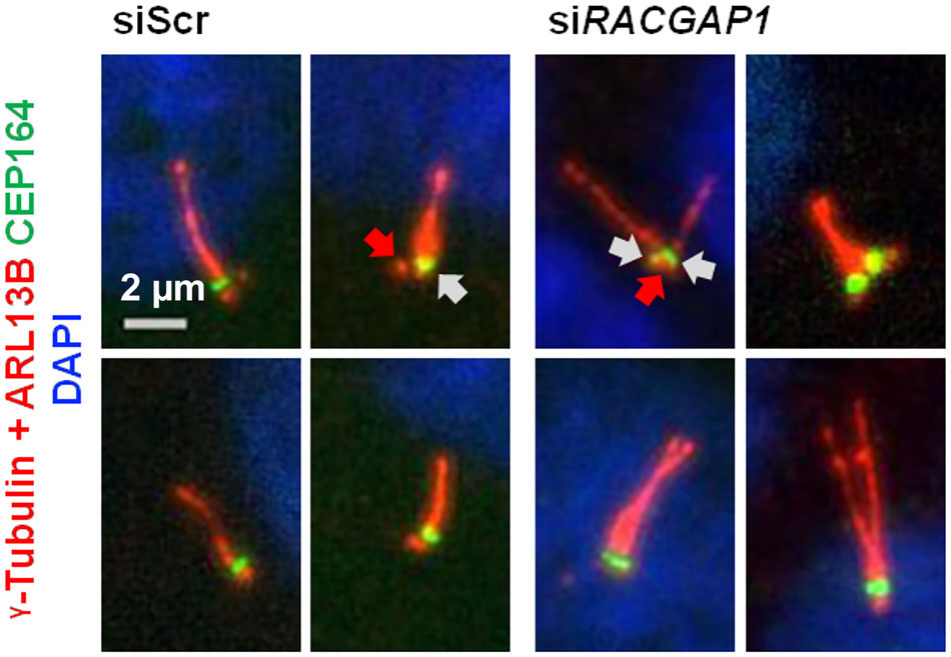
Supernumerary cilia originate from mother centrioles. hTERT‐RPE1 cells treated with scrambled negative control (siScr) and si*RACGAP1* siRNAs stained for primary cilia and basal bodies (a combination of ARL13B and g‐tubulin; red) and CEP164 (green), a marker of the distal appendages in the mother centriole. Note that siScr‐treated control cilia have one CEP164‐positive mother centriole at their base (grey arrow). Cilia in the *siRACGAP1*‐treated cells have multiple CEP164‐positive centrioles, one at the base of each cilium. Daughter centrioles do not show CEP164 staining and are indicated by a red arrow. Scale bar = 2 μm.

Whilst knockdown of *Racgap1* resulted in more than one cilium per cell, each cilium appeared to originate from an individual centriole as distinguishable at the resolution of microscopy, suggesting that there were no defects in centriole maturation or incorrect ciliogenesis from daughter centrioles. To confirm that cilia only formed from the mother centriole in each centrosome, the cells were stained for CEP164, a marker of distal appendages of mature mother centrioles that are required for ciliogenesis (Graser et al., 2007; Khanna, 2015). All cilia were found to arise from mother centrioles marked with CEP164 (Figure 4). Daughter centrioles, which do not carry CEP164, were not seen to produce cilia in any of the fields of view. This suggests that, following *RACGAP1* knockdown, centrosomes are duplicating and maturing normally prior to ciliogenesis.

### 3.5 RACGAP1 depletion in cycling cells increases the incidence of supernumerary cilia

**FIGURE 5:**
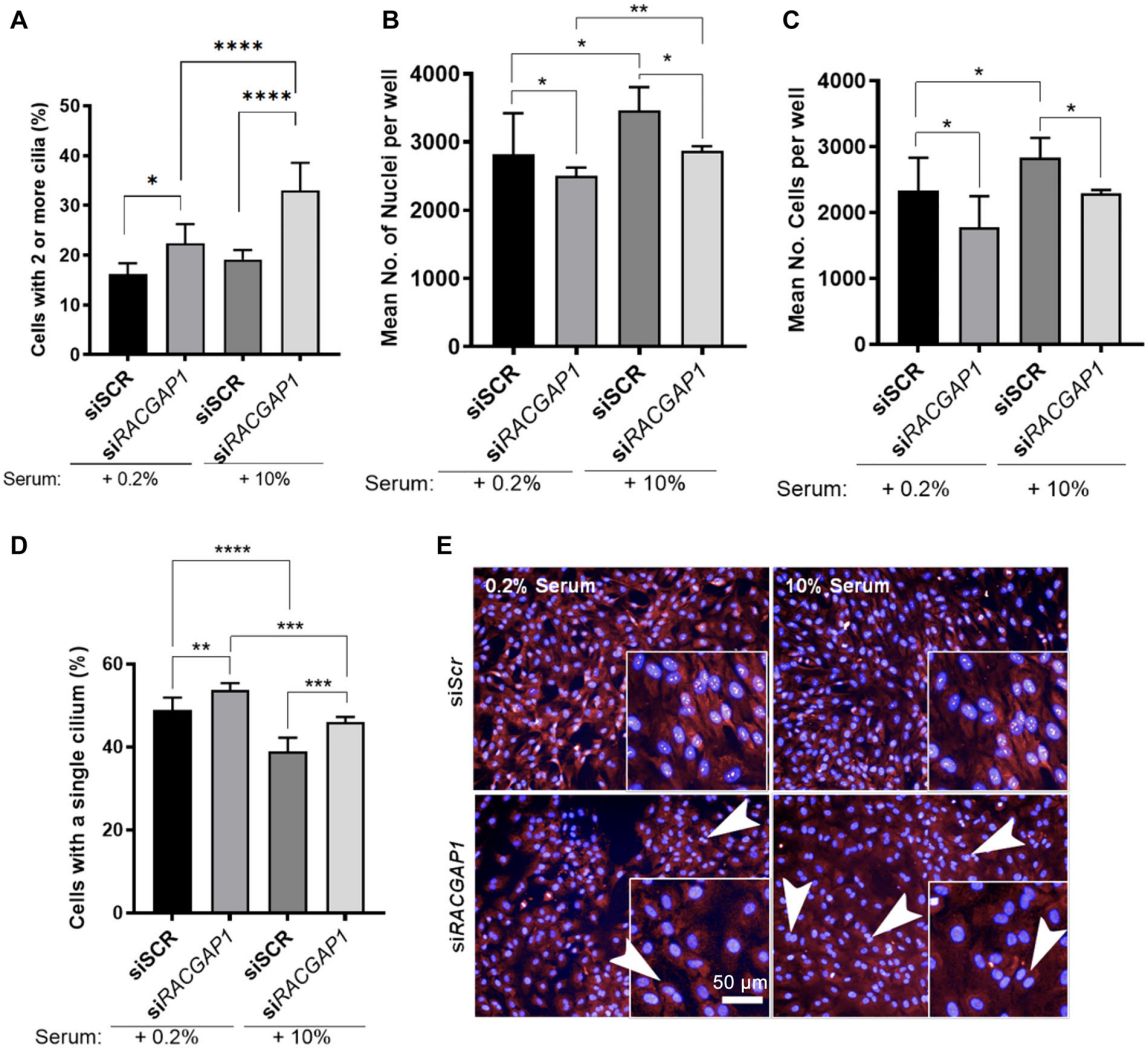
*RACGAP1* knockdown causes increased incidence of supernumerary cilia in cycling hTERT‐RPE1 cells. (a) Serum‐treated, cycling hTERT‐RPE1 cells following *RACGAP1* knockdown show an increased percentage incidence of supernumerary cilia. (b) Mean nuclei numbers in control and *siRACGAP1* knockdown cells treated with serum measured using high‐content imaging. (c) Mean cell numbers in control and si*RACGAP1* knockdown cells treated with serum measured using high‐content imaging. (d) Mean percentage of cells with a single cilium in control and *siRACGAP1* cells in normal (10%) and reduced (0.2%) serum conditions. (e) Raw image data show increased incidence of multinucleated cells (indicated by white arrowheads) in si*RACGAP1*‐treated set in 10% serum‐supplemented medium compared to 0.2% serum‐supplemented medium. Scale bar = 50 mm. One‐way ANOVA with Fisher's least significant difference was carried out for all the datasets (total of three biological replicates, >100 cells per replicate), with pair‐wise comparisons indicated by braces. Statistical significance is indicated: n.s. non‐significant; *p < 0.05; **p < 0.01; ***p < 0.001; ***p < 0.0001.

Since cells depleted for RACGAP1 had supernumerary cilia but appeared to have normal centrosome duplication and maturation, this suggested that duplicated centrosomes are being retained in an undivided cell following cytokinesis failure. These retained supernumerary centrosomes then correctly mature and mark their mother centriole with CEP164, allowing two cilia to form in the aneuploid cell. We suspected that this phenotype was a result of mitotic failure. While we could still observe cell division under the low serum conditions of our experiment, we decided to examine if the supernumerary ciliated phenotype would be more apparent in an actively cycling population of cells grown in medium supplemented with 10% serum. To test this, hTERT‐RPE1 cells were treated with either siScr‐negative control siRNA or *siRACGAP1*. For the last 24 h prior to fixation, medium supplemented with 10% serum medium was added to half of the technical replicates to observe any changes in the number of multi‐nucleated cells in induced cycling conditions. We reasoned that both the control and test cells would show a loss in cilia numbers due to ciliary resorption following addition of serum, but that cells with supernumerary cilia would increase following si*RACGAP1* treatment. As expected, hTERT‐RPE1 cells treated with *siRACGAP1* did indeed show a significant increase in the percentage of cells with supernumerary cilia after addition of serum (Figure 5a). Also, as expected this increase was larger than the one seen in the absence of serum. The number of cells with precisely one cilium decreased significantly following both siScr and si*RACGAP1* treatments, consistent with the induction of cilia resorption in the presence of serum (Figure 5d,e). There was, however, no discernible difference in cilia length following siScr and *siRACGAP1* treatments in the absence of serum. In the presence of serum, siScr‐treated cells had shorter cilia, consistent with the commencement of ciliary resorption, but this was not observed for *siRACGAP1*‐treated cells (Figure S2A). hTERT‐RPE1 cells treated with either si*SCR* control or *siRACGAP*1 increased cycling after addition of serum, as shown by an increase in nuclei number (Figure 5b). Cell numbers were unchanged, but nuclei numbers increased in *siRACGAP1*‐treated cells, suggesting that although cells were cycling, they were also becoming multi‐nucleated (Figure 5b,c,f).

### 3.6 *RACGAP1*depletion caused supernumerary cilia as a consequence of mitotic failure

**FIGURE 6:**
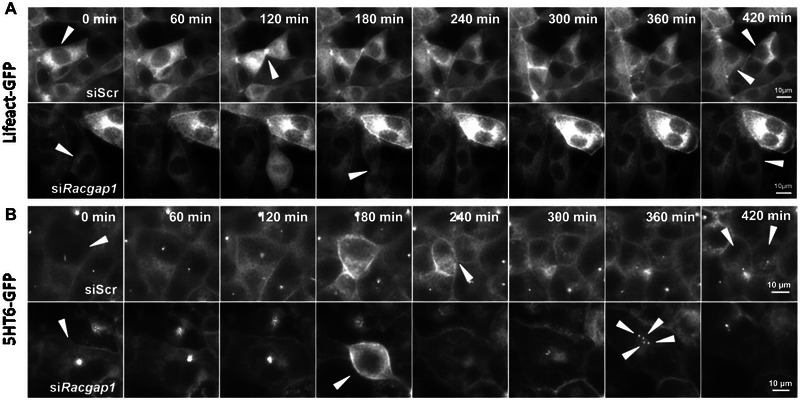
Live cell imaging of mIMCD3 cells treated with si*Racgap1* shows mitotic abscission failure. (a) Live cell imaging of mIMCD3 stably expressing LifeAct‐GFP (a biosensor for F‐actin) following si*Racgap1* and siScr control knockdowns. Note the prominent midbody visible at 120 min in the siScr‐treated control cells. Arrowheads track two daughter cells that are identifiable after successful cytokinesis from 240 min. Cells treated with si*Racgap1* display abscission failure after mitosis (180–420 min). The indicated cells (arrowheads) within the field of view are polyploid, with at least two nuclei per cell due to the mitotic failure. (b) Live cell imaging of mIMCD3 cells stably expressing 5HT6‐GFP as cilia marker, following si*Racgap1* and siScr control knockdowns. In cells treated with si*Racgap1*, arrowheads indicate a polyploid cell with multiple cilia (indicated at 360 min). Scale bars = 10 μm. The original videos for this image panel are available in the Supporting Information.

To confirm that a mitotic defect caused the supernumerary cilia phenotype, we assessed cell cycle progression using live‐cell imaging experiments. We used stably transfected lines of mouse mIMCD3 cells expressing GFP‐LifeAct, which permits visualisation of the actin cytoskeleton, or mIMCD3 cells expressing serotonin receptor 5‐HTR6‐GFP which marks the ciliary membrane. These lines permitted tracking of cell division and cilia formation in real time. Cells treated with *siRacgap1* underwent mitotic failure, causing multi‐nucleation and formation of supernumerary cilia (Figure 6a,b and Videos S1–S4). si*Racgap1*‐treated cells still appeared to ingress in telophase (Figure 6a, 180 min), but failed to complete abscission. As cells continued to cycle and additional centrioles matured, the cells became over‐ciliated with up to four cilia per cell after two failed mitotic divisions (Figure 6b). The *siRacgap1*‐treated cells were also larger (Figures 2b and S2B), likely due to errors in cytokinesis that resulted in large, multinucleate cells.

## 4 DISCUSSION

Since most cells have a single primary cilium, this study sought to identify genes that are involved in restricting formation of multiple primary cilia. We report a novel secondary downstream phenotype of *RACGAP1* knockdown in this study. To our knowledge, this is the first described link between centralspindlin complex malfunction and incidence of supernumerary primary cilia. RACGAP1 has not been previously associated with ciliogenesis or ciliary biology; however, the cytokinesis defect we observed in these cells was consistent with previous studies in eukaryotes (D'Avino et al., 2004; Yoshizaki et al., 2004). We find that supernumerary cilia in RACGAP1 knockdowns are downstream of the primary defect of mitotic failure resulting in the retention of duplicated centrosomes. These mitotic failures are likely due to a failure to assemble a centralspindlin complex, as demonstrated previously in *Caenorhabditis elegans*, *Xenopus* and zebrafish embryos with variations in *RACGAP1* orthologues (Jantsch‐Plunger et al., 2000; Miller & Bement, 2009; Warga et al., 2016). This is consistent with our observations made using live‐cell imaging, suggesting that the older retained centrioles are permitted to develop into basal bodies and to subsequently produce cilia. We observed a significant decrease in cell number in si*RACGAP1*‐treated cells. This is consistent with the finding that supernumerary centrosomes trigger PIDDosome‐dependent Caspase‐2 activation and cell cycle arrest (Fava et al., 2017).

Although *Racgap1* was the top candidate in the secondary screening, data from the original screen point to several regulators and components of the abscission machinery that support our data presented for *Racgap1* (Table S4). AURKB (Aurora kinase B), for instance, is a member of the chromosomal passenger complex (CPC), phosphorylates the centralspindlin complex being essential for its stable accumulation at the spindle midzone (Douglas et al., 2010) and functions during cytokinesis to regulate the ingression of the furrow and the abscission checkpoint. Knockdown of *Aurkb* appeared to phenocopy *Racgap1* knockdown by causing a significant increase in the incidence of supernumerary cilia in one of the replicates of the original screen (z*_2MCilia_ >+3, equivalent to *p* < 0.001). Knockdowns of other members of the CPC (*Birc5*, *Cdca8*, *Kif23* and *Anln*) also had a similar effect, albeit in only one of the screen replicates. Although these genes are plausible functional candidates, we did not take them forward in secondary screening because the z*_2MCilia_ values were not significant for both biological replicates of the primary screen. These are potential false‐negative results and highlight the importance of sufficiently stringent filtering steps used to generate the secondary screening hit list.

Furthermore, not all genes implicated in cytokinesis or abscission had a supernumerary cilia phenotype when knocked down. *Ect2* and *RhoA* are essential for correct localisation and local activation of cleavage furrow formation and the acto‐myosin contractile ring (Yuce et al., 2005). However, siRNA knockdowns of either of the genes did not phenocopy *Racgap1* knockdown. For *RhoA*, this is probably because it has multiple roles in many other cellular mechanisms including actin regulation, and acute global knockdown is likely deleterious to overall cell health.

Cells exhibiting cytokinesis failure after exiting the cell cycle return to G1 as multi‐nucleate cells with supernumerary centrioles that would normally have segregated into the daughter cell. However, there does not appear to be a checkpoint or regulatory mechanism that prevents supernumerary centrioles from maturing after being retained following mitosis failure. Subsequent to this unlicensed maturation of retained centrioles, the cells also did not appear to have any active regulation that prevented the formation of more than one primary cilium from supernumerary centrioles. Therefore, the increase in supernumerary cilia following *RACGAP1* knockdown can be explained by an increase in centrosome number following failed cell abscission, making the observed increase in cilia incidence a secondary, indirect consequence of *RACGAP1* knockdown. It is interesting to note that, from the top 10 validated hits from the secondary screen, only *Hectd2* knockdown did not cause a significant decrease in cell number concomitant with the increase in supernumerary cilia. *Hectd2* is a ubiquitin protein ligase associated with antigen processing but has been previously identified as a negative regulator of ciliogenesis (Kim et al., 2010; Shearer & Saunders, 2016). The mechanism by which Hectd2 prevents supernumerary cilia formation therefore warrants further study. More generally, negative regulators of ciliogenesis or centriole maturation could provide insights into ciliopathy disease mechanisms because centrosome amplification is currently the only described mechanism in ciliopathies that display supernumerary primary cilia (Battini et al., 2008). Since cytokinesis failure and centrosome amplification are hallmarks of several cancers (Anderhub et al., 2012; Levine et al., 2017), it will be important to analyse the contribution of supernumerary cilia to the disease phenotype.

To our knowledge, this is the first description of a supernumerary cilia phenotype due to disruption of the centralspindlin complex. Although our screen did not identify a novel pathway, the methodology used was robust in identifying hits that increased the incidence of supernumerary cilia. These results also emphasise the value of re‐interpreting large datasets from whole‐genome siRNA screens and the unexpected biological insights that can be gained from their re‐analysis.

## AUTHOR CONTRIBUTIONS

Basudha Basu and Alice V. R. Lake contributed equally to the creation of this manuscript, designed and performed experiments, analysed data, prepared figures and prepared the manuscript. Becky China performed experiments. Katarzyna Szymanska and Gabrielle Wheway completed and analysed the whole‐genome screen that this work was based on and contributed to the editing of this manuscript. Sandra Bell, Ewan Morrison, and Jacquelyn Bond supervised the research presented and contributed to the whole‐genome screen that this work is based on. Colin A Johnson led the whole‐genome screen work, supervised the research presented and advised on the writing of this manuscript.

## CONFLICT OF INTEREST STATEMENT

The authors declare no conflicts of interest.

## Supporting information

Figure S1 Information

Figure S2 Information

Table S1 Information

Table S2 Information

Table S3 Information

Table S4 Information

Video.1

Video.2

Video.3

Video.4

## Data Availability

Screen datasets are available at the following site: https://doi.org/10.5518/1044.
