# Supplementary material for: Racgap1 knockdown results in cells with multiple cilia due to cytokinesis failure
Source: Ann Hum Genet. 2023 Sep 28;88(1):45–57. doi: 10.1111/ahg.12529 (PMC10952936; doi:10.1111/ahg.12529)

**Supplementary Figure 1. STRING analysis of the final 85 hits for the increase incidence of cells with two or more cilia secondary screen**

A network formed by STRING analysis (https://string-db.org/)^14^ of the final 85 hits. The central nodes (e.g. Ccnb1, Cdk1) are involved in the G2-M transition of the cell cycle and the more peripheral nodes of the network (eg. Prim2, Tfdp1) are involved in DNA replication. Pink lines represent experimentally determined interactions, light blue lines represent interactions found in curated databases, black lines represent genes that are co-expressed, yellow lines represent genes that are co-mentioned in published abstracts.


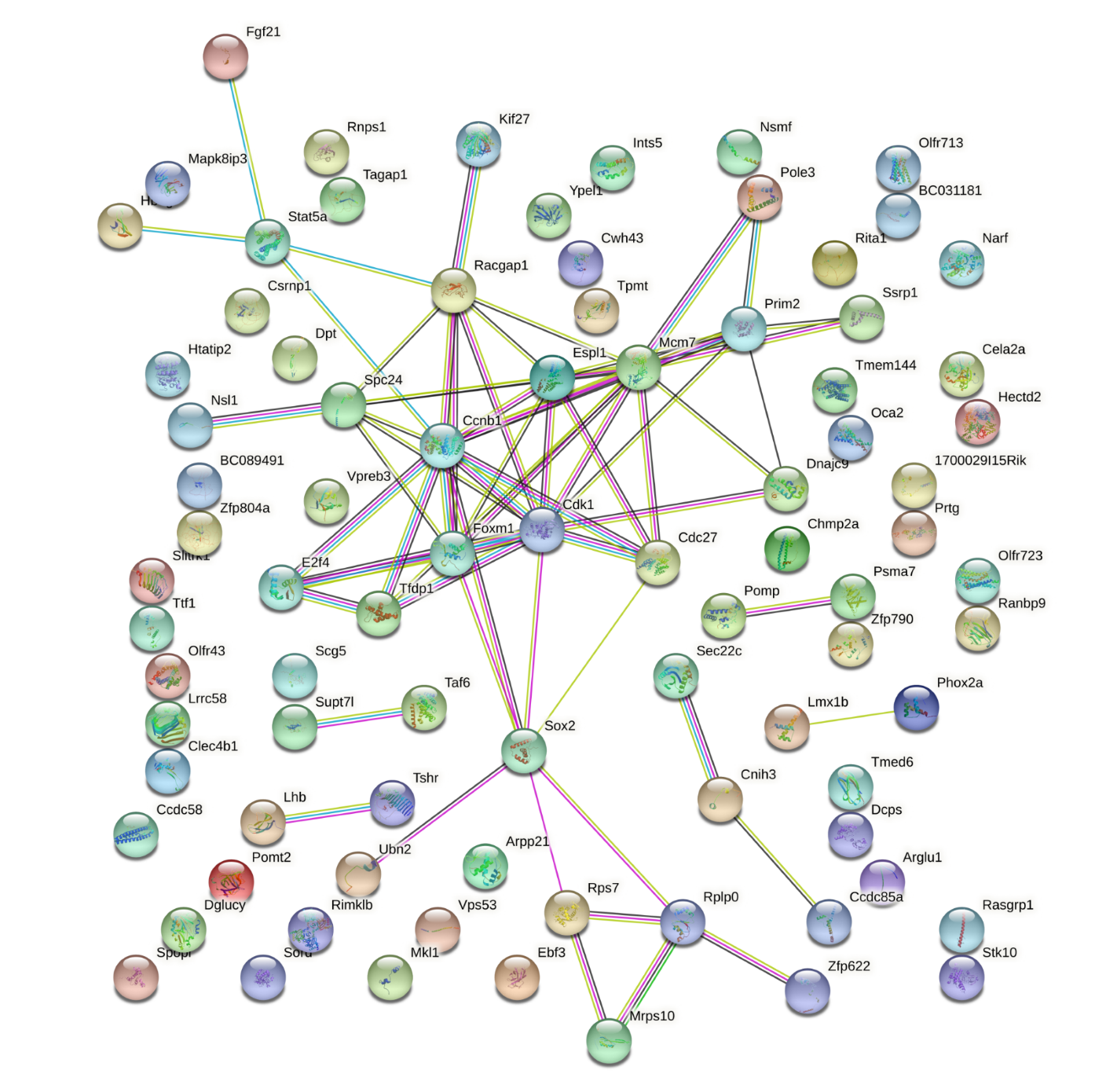

Supplement: Supplementary file 1 — Figure S1 Information [file AHG-88-45-s004.docx]
