# Supplementary material for: Racgap1 knockdown results in cells with multiple cilia due to cytokinesis failure
Source: Ann Hum Genet. 2023 Sep 28;88(1):45–57. doi: 10.1111/ahg.12529 (PMC10952936; doi:10.1111/ahg.12529)

**Supplementary Figure 2. RACGAP1 knock-down has no effect on cilia length but an increase in cell size is observed in cycling hTERT-RPE1 cells**

(A) There is no difference in cilium length between siScr and siRACGAP1 treated cells with our without serum treatment. (B) Cell area (in um^2^) in control and siRACGAP1 knockdown cells treated with and without serum measured by high-content imaging.


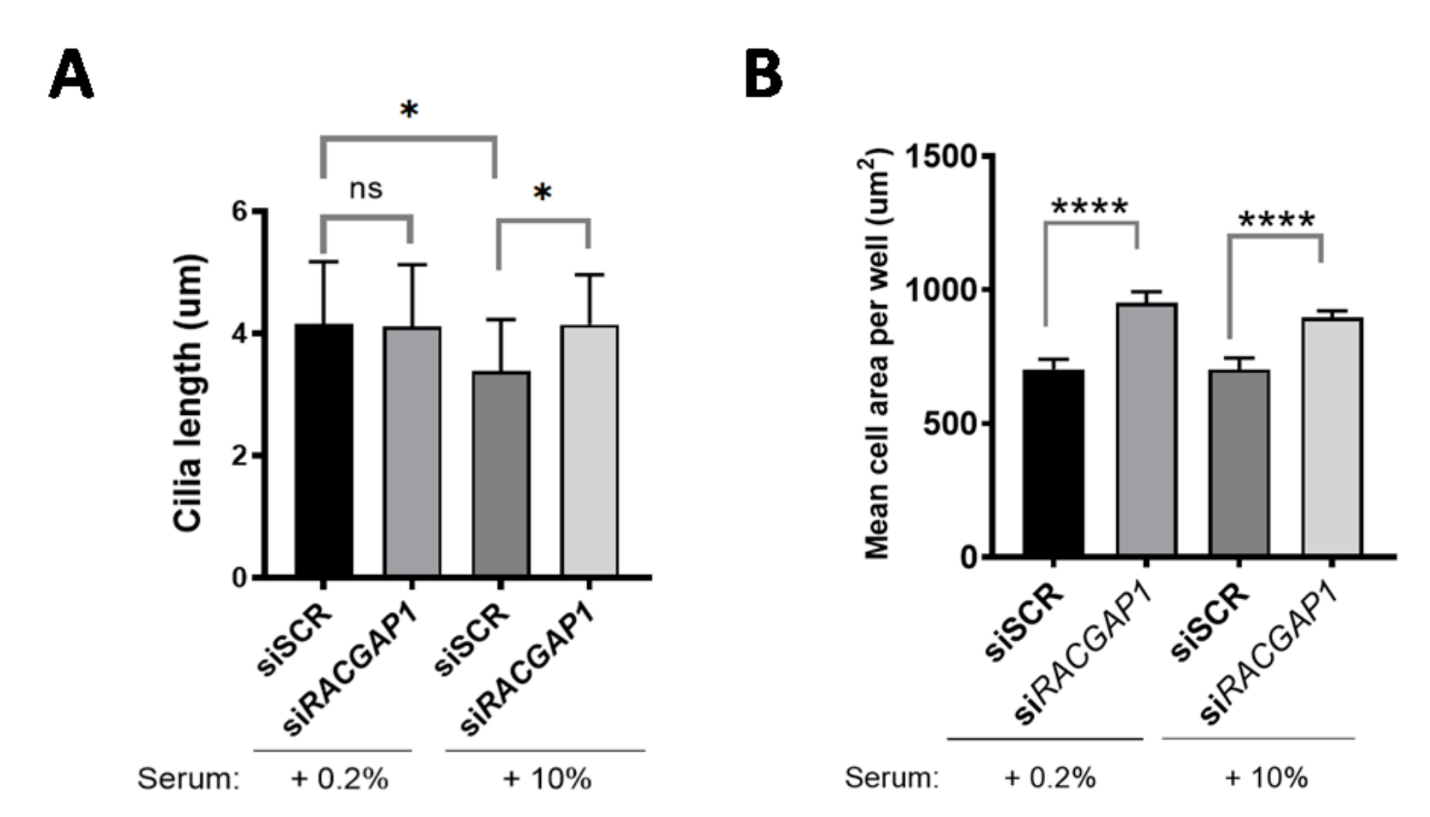

Supplement: Supplementary file 2 — Figure S2 Information [file AHG-88-45-s009.docx]
