# Supplementary material for: Racgap1 knockdown results in cells with multiple cilia due to cytokinesis failure
Source: Ann Hum Genet. 2023 Sep 28;88(1):45–57. doi: 10.1111/ahg.12529 (PMC10952936; doi:10.1111/ahg.12529)
Supplement: Supplementary file 4 — Table S2 Information [file AHG-88-45-s010.docx]

| Gene Symbol | Gene Accession | ON-TARGETplus siRNA SMARTpool Target Sequences |
| --- | --- | --- |
| 1700029I15Rik | NM_183112 | CCAACAUGGCUGACUAUUA, CAGGCCUGGUGGAUGACUA, GCAUAAAGCUAUUCUGGGC, AGCAAUCUGUUCCGGAGGA |
| Arglu1 | NM_176849 | ACAAGGAGCGGGUGCGGAA, AGGAUGAAAUUGAGCGAGA, AGAACAAGAACGACAGCGU, GAGGAACUGGAGCGGAUAU |
| Arpp21 | NM_028755 | CGACCAUACUGGAAGAAGG, GUCUGGAUGAGGAGGAGAA, GAAGCUGACCAGAAGUCUU, CAAGUCAGGAGCAGGCAAA |
| BC031181 | NM_001001181 | ACAAAUAGAAGGUCCGUGU, AGUUCUUUCUUACGAGUUU, GGGAAACAGUGCCGUCCGA, GCGAUAAGAAUAUGAGCAA |
| BC089491 | NM_175033 | GGUAAGCUCUCCACGAUUU, UGUGGGUGCUAUUGACGAA, AAGUGUUUGUGGAUGGGAA, GAAUCUAGGUCGGGUCUUU |
| Cdc27 | NM_145435 | CUAUAUGGCAAGCGCUAAA, AGGCAAUUGACAAGCGCUA, CGGCGGAAAGUGACGAAUU, CAAAUUACGCUUACGCCUA |
| Ccdc58 | NM_198645 | CUGGAUGACUUAACGUUAU, UGAAUUAAACACUACGGUU, AGUCAGAACUAAAUGUCGA, GUGAAGAGUUCGCCGAGUU |
| Ccdc85a | NM_181577 | UAUCUAAAGUGACGGACGA, CGGAAUGUCUACAGCGGCA, GCAUGUUGCUGGACGAGGA, GGCAAAGAUUGGGUCGCUA |
| Ccnb1 | NM_172301 | UAACGGAAGUUGUCGAAUU, CUGCCUCACUCUAGUUUAA, CAAACUGUGUGUAACAUAG, AAGGGUGUAUUCUUGUAUA |
| Cdk1 | NM_007659 | GAACUUCGACAUCCAAAUA, GUAUAAGGGUAGACACAGA, UGCCAGAGCGUUUGGAAUA, GGCUGUAUCUCAUCUUUGA |
| Cela2a | NM_007919 | CGAGAAACUAUGUCUGCUA, GGUGAUGGCAAGGAACUAA, UCACCAGGGUCUCCAACUA, GCCUGCUGGUUGUGGACUA |
| Chmp2a | NM_026885 | CAAAAGACCUGGUGCGUAC, GAGGAACUACUUCGGCAAA, GAACGACAGAAACUAGAAA, GGAGAUAUGUACGCAAGUU |
| Clec4b1 | NM_027218 | AGUAUAUUACCGUCGGAAU, CGAGCUGUGUGGACAAAGU, CACCAUAUGAAGAAAGUGU, GAAUGAUAUCUCUUGCAAU |
| Cnih3 | NM_028408 | GAUGCUGGCAGGUGUCGAA, UGAGGAACAUCGAACGCAU, GCAGCAGAACUGUGCGAGU, ACAUAAUCGCCUUUGACGA |
| Csrnp1 | NM_153287 | CGGAUAGCCUUGAGACCUU, CGAGUGGAAUUCAAUCAGA, CAGGUCACGUGGCCUUUAA, CCAUCUGGCUGACAUCUUU |
| Cwh43 | NM_145560 | GGACUGAUGCUAUCGGGUU, GAAAAGUGACAUCCGGCAA, GGAUUAGGGCUACGGCACA, CAACCAGAAAGUCGUCAUA |
| Dcps | NM_027030 | AGGUAGAACACGUGGCGCA, UGGCUCAAGUGAUCGAGAA, AAGCCAUCCUGAAGCGCUA, UAUCAGAUCACUUCGAGAU |
| Dglucy | NM_145448 | GUCAAGUCCAUGAGCGCUA, GGCAAUUACUACAACGCGA, GCAUGGUGAUGGUCCCGAA, GGACAAAGUGAGGCGGUGA |
| Dnajc9 | NM_134081 | CUACAGCGCCUUCGUCAAA, AAGAAGAGCUAAACGAUAU, GGUCUUAAUCCUACACGGA, AGACAAAAGGAUCGGCAAA |
| Dpt | NM_019759 | AAUCAGUGCUGGAUCGUGA, GGGUGAAUCUUAACCGCCA, GCGAAUUCGAAAACGUUUA, AGUACCAGGACUACGGUGA |
| E2f4 | NM_148952 | GCAUCGGUCUGAUCGAGAA, GCACUGCUGGAUAGUAGUA, UCUGUGAUCUCUUUGAUGU, CUACGUGACUCAUGAAGAC |
| Ebf3 | NM_010096 | GCAGCCAGCUAGCCGUUAA, CUUAAUGGCUCCUCCGCUA, GGGCUUAGCUACACGAACA, CCAAUGACCAAGUCGGCUA |
| Espl1 | NM_001014976 | CUAGUAACUUGGAGGAAUU, GGUAAUCUCUGGGAUGUGA, GAUGUGCUCUCCAUUCAGA, UCACCGGGAUUGUGUAUGA |
| Fgf21 | NM_020013 | GUCAAAGCCUCUAGGUUUC, CUACACAGAUGACGACCAA, UGGAUGAGAUCUAGAGUUG, GAGCAUGGUAGAGCCUUUA |
| Foxm1 | NM_008021 | GAGCAUCAUCACAGCGCUA, CUGGAGCAGAAUCGGGUUA, GCGCACGGCGAAAGAUGAA, UGUGAAAGCCUAUUGGAUU |
| Hbegf | NM_010415 | UGCUGAUGUUAGCGUGUAA, CUGAGACAGUGGUUCGUUA, UGAAAUACCGCAAGCUCGA, UGGAUUAGAGUGUCAGCUA |
| Hectd2 | NM_172637 | GCACACAGUACGACGGCUA, UGUCAGUACCCAUUCGUUA, GGACACAUCUGGUUAGCGA, CUACACAGCAGCCGAAGAA |
| Htatip2 | NM_016865 | GAGAUUAUGUGCUCAAGUC, GAUCGACUCUCAGUGUUUC, CAAAGUAACGCUCAUUGGU, GUCCAGCAGUUUCUUAUAC |
| Ints5 | NM_176843 | GGGUAAUAUGCACGAGGUA, CAUUACGGCUAGAACGGAA, GACGGGAACUGUUGCGCAU, CUUCUAAGUCCGUACGUAA |
| Kif27 | NM_175214 | GCGAGAAACGGAACGUAAA, GCUUGUACAUUGAACGAUU, CAGAAAGAGUUCCGUAAAA, GCAGAGCAUUGCCGAUAAA |
| Lhb | NM_008497 | CAGCAGGUGUUCUAAUCAU, CAGCAUCUGUGCCGGCUAC, UAGCAUGGUCCGAGUACUG, CCUCCCUCCUUCCCAAUAA |
| Lmx1b | NM_010725 | GCGAGAGGCAACUGCGCAA, CAGUGGAGAUGACGGGAAA, GCGAAGAGCUUUCAAGGCA, GGAAGGUCCGAGAGACAUU |
| Lrrc58 | NM_177093 | ACCAUUAAGAUCCGAAGUA, AAAGUGUGGUGGAGUCUAU, UUACCUGCCUCGAGAGAUC, CCCUCAGUCUGCACAAUAA |
| Mapk8ip3 | NM_013931 | GAUCCAAGAUGCAGCAAGU, GAGGUGGACUGAAAUGAUC, GCUACCCGCUGCAAUGUUC, CAACACUGACUCCUUGUAU |
| Mcm7 | NM_008568 | GCUCCUAUCUUACAUCGAC, UGACAAAGAGUGACGAUGA, GGAGAUCUAUGGACAUGAA, GGACAUGAAACUUAUGAGA |
| Mkl1 | NM_153049 | GGACCGAGGACUAUUUGAA, GCUGCGUCCUGCUGUCUAA, GGUCAGCUCUUGUAACAGC, GCACAUGGAUGAUCUGUUU |
| Mrps10 | NM_183086 | CCGAUGAGCCAGACACGUU, GGAUGCUUGUGUCCCGAUA, GAGUAUAUCCAGCGAAACU, AAACAUUUGUGUAGCGCUG |
| Narf | NM_026272 | GCGUACGGCUUUCGCAACA, GAUAUCAAGUGGUGACAAA, GCGUCAUGAUGGAGUGAGU, CGAGAGGGACUUUCCACUA |
| Nsl1 | NM_198654 | UCUCUUACCUGGUGCGUCA, GCUCUGCAUUUCAGCGUGA, CAGCAGGCCUGUGGUAAUU, GGAAUACGGCUGAGUGCCU |
| Nsmf | NM_020276 | CACUGAACAAUGUACGAUA, GGGUGAAGGCCCAGACGUU, CCAAGGUGCCAAAGGCCGA, UGUCCUCGGUAGCGGCCAA |
| Oca2 | NM_021879 | GAUCUUAGUAGCCGUAUUU, CUGUAAAGGCAUAUCAACU, GAACAGACUGCCCUACUAA, GUGGAUUGAUUUCGAGACU |
| Olfr1447 | NM_146703 | CAUGAAUGGGCUACUAAUA, ACAAAUUCCUCCAUCCAUA, UAAUCAAGUUAGGGAACAU, CCAUCAUGACUAAAAGUAU |
| Olfr43 | NM_146711 | CAGGAAUAUUUCUUCUUCA, GGUGAUUGCAAAUGCUAAU, CAUUGAUAACUGUGAUGUA, CAGUGGUGUCCUUGUAUUA |
| Olfr652 | NM_147048 | GCAAUUAUCAGCAGGAGCU, CCAUAUACUGUUUGCAAAU, CCAUGUGUGUUCUUGCUAA, UGGCUUUGCAGUACCAAUA |
| Olfr713 | NM_147034 | GCAAGAAAUUGUUAUCGUU, CAGCACAGUUUGAAGUCUA, UGGCUUCAAUCUAGUCAUU, UCAGAAACCAUAUCACAUA |
| Olfr723 | NM_001011530 | CAACAAGUCAGCUGCACAU, GGGCAACAUCCUUAUUAUA, UGAAAUGACCCUUUUGAUA, CCUAUUAUUUACACACUGA |
| Phox2a | NM_008887 | CAUUUACACUCGCGAGGAA, UCAAGGAGUUGGAGCGCGU, CUGAAGAGUCCCACCGCCA, GCGCAGAGGUUAGACACUA |
| Pole3 | NM_021498 | AAGAGAGACUGGACGAGGA, GGAAAGCGCAAGACUCUCA, CCUGAGAUCCUGAGACAUG, GUGCCUGAGAUCAAAAUAA |
| Pomp | NM_025624 | CAGUCACCCUCUCGAGUUA, UCACGAUCUUCUCCGGAAA, ACGUGAUGGUGGAACAUAA, CCAUUGGUUUUGAGGAUAU |
| Pomt2 | NM_153415 | GAGUCUAUCUGCUCGGCAA, GGGCCAAUGACACGGACUU, GCGUAUGGCUCUGUAAUCA, CAAAGAAACUACUCGGAAC |
| Prim2 | NM_008922 | GCAAGAUUUCCUUAGAUCA, UCUUACAAUAUCCGGCAUA, ACAUAUCGUUGACGGAGUU, GAGUAUGAGCCACGGCGAA |
| Prtg | NM_175485 | CUUUCUACAUUGUGGCGUA, GGAGCAAAAUUGUCCGAAA, GUGUGGAGCUUUAGCGAAC, AAACAGAUAUAGACUCGAA |
| Psma7 | NM_011969 | AGUCUGAAGCAGCGUUAUA, ACAUCGAACUUGCCGUCAU, GCGCAGGCCAUUUGGUAUC, GGUCCAGUCAGGUGGCAAA |
| Racgap1 | NM_012025 | CCAGGCAAAUGAAUCAAUA, UGACACAUCUGGCAGUAUU, GGACACCGGUUAAGAUUGG, GUAAUCAAGUGGACGUGGA |
| Ranbp9 | NM_019930 | GAAGACUACAUGCGAGAAU, GAAGAUUGUGACACCGAAA, CUAAACAUGACCACGAAAU, GGUCACAGCAAGUUAAUAA |
| Rasgrp1 | NM_011246 | CAAAUUAAUUCUCGAGACU, CUUGUAAAUAGCUGCGUAA, GAGAGAGGCUCCGCGGAAA, UCAAUAAGGUUCUGGGCGA |
| Rimklb | XM_981743 | GGAUAUAUGCUCUGUUAAU, CUGCGAGGCCAAUGCAAAU, AGGAUUAUUUGGAGUCAUA, GUAUAUAUGUCGCAUGCAA |
| Rita1 | NM_029096 | GUGCAGAAUUGUAACCGAU, CCUCCUUGGAAGUGAGAUU, CCUCACACCAAGGAAGAAA, CGAAGGGCUCUCUGGCCAA |
| Rnps1 | NM_009070 | GCAGGGACAUUGAUUCGUA, GCUCCAACUCCUCCCGAUA, GUUUGAGAAUCCCGAUGAA, GGAAAAGGCGGCACGUUCA |
| Rplp0 | NM_007475 | GGACAAAGAUUUUCCAGGU, AGAUGGAAAUAAAGGCUUA, CGCCAAAGCAACCAAGUCA, GAGGAAUCAGAUGAGGAUA |
| Rps7 | NM_011300 | CGGAAAAGCCGUACGAAAA, GCGAGAAGCCGGACGAGUU, AGUUGGUGGUGGUCGGAAA, CAGCAGUGCACGACGCCAU |
| Scrambled | N/A | UGGUUUACAUGUCGACUAA, UGGUUUACAUGUUGUGUGA,  UGGUUUACAUGUUUUCUGA, UGGUUUACAUGUUUUCCUA |
| Scg5 | NM_009162 | GAUGAUGGAUGUCUAGAAA, CCAAAUCACUGUCCUCUUG, GGUUGAUGUUUGAGUGGAA, GGAACAAGAAACUCCUUUA |
| Sec22c | NM_178677 | GCAUCGUACGGGUGAGGGA, CUUCAUAGCUUCCUACGAC, GGCCUUACGCCUUUCUUGA, UUACUACGCCCAGGAGUUU |
| Slitrk1 | NM_199065 | UGUAACAACCGGAACGUGA, UGGAGUACAACGCGAUUCA, UCACAAGUCUGCAGCGCUU, CCGAGGUGCUGAUGAGCGA |
| Sord | NM_146126 | UCUCAAACUCGUCGCAUAA, GCUAGAAGGGGAAGCCGUA, GCACGCAGCCAUUCGGGAA, GAUGGGUAUCACAGCGUAA |
| Sox2 | NM_011443 | GAAGAAGGAUAAGUACACG, GCACCCGGAUUAUAAAUAC, GCUCGCAGACCUACAUGAA, GGACAGCUACGCGCACAUG |
| Spopl | NM_029773 | GGGAUUAGAUGACGAAAGU, GUGCAGUGUUCUUCGACAA, GAUGACAAGCUUACGUUAU, UGAAUGAGUGGGCCGAUUA |
| Ssrp1 | NM_182990 | GCGAGAAGAUCAAGUCGGA, GCGUACAUGCUGUGGCUUA, CGAAUCGGAGUUUGAGAAA, GCAUUAGGCCAUGGGCUUA |
| Stat5a | NM_011488 | GCAACGAGCUGGUGUUCCA, GGACCGAAACCUCUGGAAU, UGAACUACCUUAUCUACGU, GAACACGUAUGACCGCUGU |
| Stk10 | NM_009288 | GGCAUGACCUGCUGCGUAA, CAAAGAAACGGGCUCAUUG, AAAGCGAGACUCCUUCAUA, GCAAUAAGGCUCUUCGGGA |
| Supt7l | NM_028150 | GAACCUGGCUCACGUGAAA, AUGCAAUAUAUGACGUCUA, GCGCUUUGCUGUCGACCGA, AGGCAAUGUGUCUGCGCAU |
| Taf6 | NM_009315 | CGACAGAAGCUCACUACCA, UGAGCAACAUCGACCGUAU, GAUGUGAUUAAGACGCUAA, AGAGCUGGGUCGACGAGAA |
| Tagap1 | NM_147155 | AGCUCAGAGUCCAGCGUUA, CAACAAGACAGGCGGUUCU, ACGAAGAGGCCAUUUAUUA, CGACAACACGUUCCAGUUA |
| Tas2r106 | NM_207016 | GCAGAAUCUUUGUCAUAUG, UCUCAACCUGGGAGUUAUA, GGGUAAUUAUCAAUCAUAC, GAUAGAAACAUUGAGCUUU |
| Tfdp1 | NM_009361 | CGUUUGAGAUCCACGAUGA, CGACGAGGAGGAUUGAUUA, CAAACGAAUCAGCUUAUGA, AGAAGGAGAUCAAAUGGAU |
| Tmed6 | NM_025458 | GCUACGAGGUUCAGCGCAU, ACACUCUGGAUGCGAUUAA, CCGUGGAGCUGAUCGGAAU, UCGAAUGUUUCUGGCAAUU |
| Tmem144 | NM_027495 | CAACAUUGCUGUCGUUCCA, CUGGAAUACUCUACGGAUC, UAGACUACGUGUUCGCACA, GCUCAAAGGCAUAUCGGAU |
| Tpmt | NM_016785 | AGGCCAGAGUGGACUGCGA, GAUGAAAUGGUUCGCAGAU, GGUGUGGAAAUCAGUGAAA, GGCUAUCAAUCCAGGCGAU |
| Tshr | NM_011648 | CAGUACAACCCUCGAGAUA, ACCAGAAGCUUAACCUAUA, AGGAUGAGGUCGUUGGUUU, CCAAGUCGGACGAGUUUAA |
| Ttf1 | NM_009442 | GCGUGAAACAGGAGAGCUG, GGAUAUGGAAACUGGGAUC, CCUUCUAGCUGUUCCCAUA, CAUAGUUCCUCCAGCAUAU |
| Ubn2 | NM_177185 | GAACACAGGGUGCUACUAA, ACACUAAGUUACCACGGAA, GUAAAGAAGCGGAAGCGGA, ACUCAGAGGCUUAUGACGA |
| Vpreb3 | NM_009514 | CGGAGGAUGAUGCCGAUUA, ACUGUUAGAUCCUGUGUAA, CUAUUACGCAGAAGAGGAA, CCCGACAGAUUCUCAGCUA |
| Vps53 | NM_026664 | GAGCCUGACUGAACGAAUU, CUGAGAACAUUACGGUUAU, AAAGAGAUCACCCGAGAUA, GAGUGGUGCAUGACGGAGA |
| Ypel1 | NM_023249 | GGUAAAGUGAAGCGGUCCA, GAUUGGAAGGCUGGUAAUA, UGAUGUAGAAGGCGAGUGA, UUGAAUCCCUGUCGUGUAA |
| Zfp622 | NM_144523 | CAUAAGGUGGGAAACGCAA, GAUACUACAAACAGCGAUU, GGUCAAAGCUAGGACGUUC, UCGAACAGCAGGCGAAGAA |
| Zfp790 | NM_146185 | ACAAACAGCACGUGCGCAA, GUAAGAAAUCACACGAAUU, GGUAAGAAGCUGUACGAGU, CCUCAGACCUCUCUCGACA |
| Zfp804a | XM_001000810 | AGAGAGAAUUUGCUCGAAA, CAAAGAAAGCCACGGUGAA, ACUACAAACAGUAACGAAA, GGAGAUGGAACUACGAAAA |

### Suppl. Table 2. siRNAs used in the secondary screen for increased incidence of cells with two or more cilia (supernumerary cilia) in mIMCD3 cells.

These siRNAs were purchased from Dharmacon™ as an RNAi Cherry-pick Library, 0.1nmol stocks, in 96 well plate format for screening. All siRNAs targeted the mouse orthologue of the genes listed.
