# Supplementary material for: Racgap1 knockdown results in cells with multiple cilia due to cytokinesis failure
Source: Ann Hum Genet. 2023 Sep 28;88(1):45–57. doi: 10.1111/ahg.12529 (PMC10952936; doi:10.1111/ahg.12529)
Supplement: Supplementary file 5 — Table S3 Information [file AHG-88-45-s006.docx]

| Gene Symbol | Gene Accession | ON-TARGETplus siRNA SMARTpool Target Sequences |
| --- | --- | --- |
| RACGAP1 | NM_013277 | UAAAUGAGAUUGAGCAAAG, GCGAAGUGCUCUGGAUGUU,  CCACAGACACCAGAUAUUA, GAAGUCACAUCUGCCUGUU |
| Scrambled | N/A | UGGUUUACAUGUCGACUAA, UGGUUUACAUGUUGUGUGA,  UGGUUUACAUGUUUUCUGA, UGGUUUACAUGUUUUCCUA |

### Suppl. Table 3. siRNAs used in the validation experiment for increased incidence of cells with two or more cilia (supernumerary cilia) in hTERT-RPE1 cells.

These siRNAs were purchased from Dharmacon™ as 5nmol stocks, in tube format. All siRNAs targeted the human orthologue.
