# Supplementary material for: Racgap1 knockdown results in cells with multiple cilia due to cytokinesis failure
Source: Ann Hum Genet. 2023 Sep 28;88(1):45–57. doi: 10.1111/ahg.12529 (PMC10952936; doi:10.1111/ahg.12529)
Supplement: Supplementary file 6 — Table S4 Information [file AHG-88-45-s007.docx]

|  | **Run 1 z*-score** | **Run 2 z*-score** | **Z*_2Mcilia_** |
| --- | --- | --- | --- |
| *Racgap1* | 3.637513 | 3.931008 | 3.784261 |
| *Birc5* | 5.078846 | -0.14774 | 2.465552 |
| *Cdca8* | 3.727382 | 1.912464 | 2.819923 |
| *Kif23* | 5.556383 | 1.014768 | 3.285575 |
| *Anln* | 3.826827 | 1.452181 | 2.639504 |
| *Aurkb* | 4.975328 | 1.896057 | 3.435692 |
| *RhoA* | -1.13110 | -0.58016 | -0.85563 |
| *Ect2* | -0.76198 | -1.15997 | -0.96097 |

**Supplementary Table 4. Whole genome screen data of genes that are components of the centralspindlin and abscission machinery**

The 2 components of the centralspindlin complex (*Kif23* & *Racgap1*) when knocked down by siRNA cause a significant increase in supernumerary cilia, as shown by the average robust z-score for incidence of cells with 2 or more cilia (Z2MCilia). *Aurkb* knock-down shows a similar effect. Both *Kif23* and *Aurkb* were not taken forward for secondary screening because they failed to meet the criterion that required a significant increase in cells with supernumerary cilia in both Run1 and Run2. Green represents numbers greater Z*2MCilia >1.96 and red represents numbers <1.96.
